# Blood Lead and Water Treatment: Miranda et al. Respond

**DOI:** 10.1289/ehp.10453R

**Published:** 2007-10

**Authors:** Marie Lynn Miranda, Martha Keating, Dohyeong Kim, Andrew P. Hull, Christopher J. Paul, M. Alicia Overstreet Galeano

**Affiliations:** Nicholas School of the Environment and Earth Sciences, Duke University, Durham, North Carolina, E-mail: mmiranda@duke.edu

In response to our study (Miranda et al. 2007), in which we found an association between age of housing, water treatment practices, and blood lead levels, Weintraub concludes that

a more prudent approach to prevent lead exposure via drinking water is that municipalities ensure careful corrosion control and remove lead service lines and distribution pipes, regardless of the method of residual disinfectant used.

Unfortunately, this approach has fallen notably short as evidenced by tests of residential tap water in Washington, DC (Edwards and Dudi 2004), Greenville, North Carolina (Renner 2005), and in 2006, in Durham, North Carolina (Biesecker 2006). Each of these cities had increased levels of lead in tap water following a change in water treatment practices necessitated by a switch to chloramines for disinfection.

We stand by our hypothesis that the effect of chloramines on blood lead levels would be less important in newer housing stock. Weintraub cites a U.S. Environmental Protection Agency Fact Sheet (U.S. EPA 1993) as evidence that older buildings would pose less, not more, of a lead risk because of the protective effect of mineral deposits coating the inside of the pipe. However, as the U.S. EPA noted, lead levels decrease as a building ages only “if the water is not corrosive.” An increase in the corrosivity of treated water after a switch to chloramines may expose lead that was shielded by mineral deposits (Schock 1990). This supports our reasoning that newer housing without lead service lines or lead solder and low-lead fixtures would pose less exposure risk.

We did not use an ecologic study design in our analysis. Our outcome variable was an individual measure of blood lead level, and our explanatory variables were either individual or census-level measures. We did not attempt to infer an individual-level association of variables from an association demonstrated only for aggregated variables.

We coined the phrase “Wayne Water Systems” to represent the collection of water systems in Wayne County, North Carolina, that do not use chloramines. “Wayne Water Systems” includes the five water systems that make up the Wayne Water Districts, as well as the five other active community water systems (Fremont, Fork, Pikeville, Mt. Olive, and Southern Wayne) in the county. We should have noted this in the text.

With respect to the categorical analysis, stratifying housing age by 25-year categories did not lead to misclassification of the housing, in spite of grouping together houses built before and after the ban of lead solder in 1986. We performed model iterations with several different age categories of housing and presented the best-performing model. We did run a model with housing built after 1986 as a referent group, but we found the variable of 1976–1986 statistically insignificant.

We based our finding that 15.6% of the housing stock in Wayne County was built before 1925 on tax parcel data, which we consider to more accurately represent housing age than homeowner responses to age-of-housing questions posed by the U.S. Census. We would have preferred to include all blood lead records, but it was not possible because we could not geocode incomplete addresses. However, when we compared the blood lead levels of children from geocoded and nongeocoded addresses, we did not find a significant difference. Records of children from Seymour Johnson Air Force Base were included in the analysis.

In July 2006, the U.S. EPA proposed changes to the Lead and Copper Rule that would require water systems to notify, and obtain approval from, state regulatory agencies before changing a water treatment process (U.S. EPA 2006). As the U.S. EPA noted, if a water system notifies the state after changes have already been made, there may be little or no opportunity to minimize any corrosion problems to prevent lead from leaching from plumbing components. We suggest the use of housing age as a simple metric for states to use in designing a water monitoring program, and expanding blood lead screening in advance of changes in water treatment practices.

Weintraub states that our recommendations “to exclude certain children” based on water disinfection practices could have “substantial unintended impacts.” This is a misrepresentation of our recommendations. Based on the Wayne County results (and those in Washington, Greenville, and Durham), we recommended that local health departments expand their scope of targeted screening in the years following the introduction of chloramines as a supplement to, not a replacement of, existing programs that are in place to provide blood lead screening services to children at risk of lead exposure. We also suggested that health departments might target more intensive outreach and education to residents of older housing, a strategy that would help focus limited resources on residences where additional lead sources may also be present.

Our recommendations are consistent with the U.S. EPA’s rationale for revising the Lead and Copper Rule to require advance notification of water treatment changes, and with recommendations by water chemistry experts to closely monitor water lead levels after changing to chloramines for disinfection.

## References

[b1-ehp0115-a00488] Biesecker M (2006). Durham lead fear widens. News and Observer (Raleigh, NC), 13 June. http://www.newsobserver.com/news/health_science/lead/story/450089.html.

[b2-ehp0115-a00488] Edwards M, Dudi A (2004). Role of chlorine and chloramine in corrosion of lead-bearing plumbing materials. J Am Water Works Assoc.

[b3-ehp0115-a00488] Miranda ML, Kim D, Hull AP, Paul CJ, Galeano MA (2007). Changes in blood lead levels associated with use of chloramines in water treatment systems. Environ Health Perspect.

[b4-ehp0115-a00488] Renner R (2005). Chloramines again linked to lead in drinking water. Environ Sci Technol.

[b5-ehp0115-a00488] Schock M (1990). Causes of temporal variability of lead in domestic plumbing systems. Environ Monit Assess.

[b6-ehp0115-a00488] U.S. EPA (Environmental Protection Agency) (1993). Actions You Can Take to Reduce Lead in Drinking Water. EPA 810-F-93-001. http://www.epa.gov/safewater/lead/lead1.html.

[b7-ehp0115-a00488] U.S. EPA (Environmental Protection Agency) (2006). National primary drinking water regulations for lead and copper: short-term regulatory revisions and clarifications; proposed rule. Fed Reg.

